# Editorial: Radiation and the Immune System: Current Knowledge and Future Perspectives

**DOI:** 10.3389/fimmu.2017.01933

**Published:** 2018-01-23

**Authors:** Katalin Lumniczky, Serge M. Candéias, Udo S. Gaipl, Benjamin Frey

**Affiliations:** ^1^Radiation Medicine, National Public Health Institute (OKI), Budapest, Hungary; ^2^Université Grenoble Alpes, CEA, CNRS, BIG-LCBM, Grenoble, France; ^3^Radiation Oncology, Universitätsklinikum Erlangen, Erlangen, Germany

**Keywords:** radiation, immune system, inflammation, immunotherapy, tumor immunity, bystander effects, low dose irradiation, biomarkers

**Editorial on the Research Topic**

**Radiation and the Immune System: Current Knowledge and Future Perspectives**

At present, the opinions about the interaction between ionizing radiation and the immune system are largely controversial. For long, high-dose ionizing radiation was considered as net immune suppressing mainly due to the exquisite radiosensitivity of the lymphoid system. While this increased radiosensitivity cannot be contested, a rapidly growing number of scientific publications have demonstrated a very heterogeneous quantitative and functional response of the different components of the immune system to radiation (1–3). A major milestone was achieved in the field of onco-immunology, where it has been shown that local tumor irradiation could modulate the immunogenicity of tumor cells and also the antitumor immune responsiveness both locally in the tumor microenvironment and at a systemic level (4–6). This latter observation opened the gate for studies exploring optimal combinations of radiotherapy (RT) and immunotherapy in order to achieve a synergistic effect.

It was additionally recognized that some of the radiation-induced late side effects are of immune and inflammatory nature. At present, one of the most studied fields of research in radiation biology is focused around the biological effects of low doses, where the observed pathophysiological endpoints are due to mechanisms other than radiation-induced direct cell killing. Such mechanisms are for example radiation-induced bystander effects or abscopal effects, which by sharing common mediators with various immunological signaling processes, are most probably immune-mediated.

The multitude of studies investigating the interactions between ionizing radiation and the immune system lead to the emergence of a new, highly interdisciplinary scientific field called radioimmunobiology, with the potential to induce a paradigm change in this area and to achieve direct clinical applications within a relatively short term. Scientific primary and overview papers collected in the present Research Topic aim to give an up-to-date state of the art of the complex interactions between the immune system and ionizing radiation while highlighting future perspectives as well. This paradigm change is nicely illustrated in the review by Schaue, who gives a comprehensive historical overview regarding the interaction between ionizing radiation in general and RT, in particular, and the immune system.

Although the cell autonomous effects of ionizing radiation are well established, there is nowadays growing evidence that intercellular communication plays a major role in the outcome of radiation exposure at the tissue level. This is especially true in cancer therapy. Radiation exposure aims at killing tumor cells, but efficient tumor control/eradication also requires the activation of the immune system. The tumor microenvironment indeed contains various subsets of immune cells, both myeloid cells and lymphocytes. Among the myeloid cells, myeloid-derived suppressor cells and tumor-associated macrophages provide a supportive environment for tumor growth, in part by suppressing the activity of cytotoxic T cells. Wennerberg et al. summarize further immune suppressive properties of radiation on the cellular and molecular level besides the impact of radiation on expression of inhibitory immune checkpoint molecules such as PD-L1. The impact of RT and radiochemotherapy (RCT) on the latter on tumor cells of different tumor entities is the focus of the research article by Derer et al. They show that, in particular, RCT increases the expression of PD-L1 on melanoma and glioblastoma cells. This demands radioimmunotherapies (RIT) to counteract radiation-induced immune suppression. For the design of beneficial RIT, many additional parameters have to be taken into account. What is the best radiation dose and radiation quality? Ebner et al. review the unique biological and physical benefits of particle irradiation that may be superior in some aspects for the generation of systemic radiation-induced immune-mediated effects. Besides the radiation quality, the dose and the chronological sequence of immune cell infiltration into the tumor has to be kept in mind. Frey et al. present data on timely restricted immune cell infiltration following hypofractionated radiation. Cytotoxic T cells follow the antigen-presenting cells and are present for only 2 days. Here, re-irradiation of the tumor should be revisited to spare the immune cells, and boosting of the immune system at this time point could be particularly effective. Since the inflamed microenvironment of tumors impacts on growth and progression, several articles deal with modulation of inflammation by radiation. Rödel et al. review radiation-induced mechanisms contributing to a modulation of proliferative and inflammatory processes. They focus on summarizing innovative concepts of treating hyperproliferative diseases by low and moderate doses of ionizing radiation. Since inflammatory events and bone metabolism are interconnected, radiation also impacts on bone turnover. Cucu et al. demonstrate in serum samples of patients who were exposed to very low doses of alpha-irradiation in radon spa that collagen fragments (in particular CTX-I) are decreased after radiation exposure. This suggests a reduced bone resorption by osteoclasts. The interleukin IL-33 has been described as an intracellular alarmin being involved in many inflammatory processes. Kurow et al. revealed that the release of full length IL33 in the damaged tissue does exacerbate radiation-induced skin reactions.

Thus, radiation acts and modulates immune reactions, including inflammation, at various levels and in all tissues. Immune cells and, in particular, lymphocytes are key players in the response against radiation-modified tumor cells. In order to highlight transcriptionally responsive genes, which play a role in the inflammation response, Manning et al. monitored the expression of about 250 genes associated with the inflammation response over the course of the RT in blood of patients with endometrial or head and neck cancer. Some of these inflammation-related genes could be promising biomarkers of radiation exposure and susceptibility to radiation-induced toxicity. Radiation-induced inflammatory reactions are also heavily involved in the development of radiation-related side effects in various tissues. Several papers within this research topic specifically focus on this aspect. Wirsdörfer and Jendrossek summarize radiation-dependent mechanism of acute and chronic environmental lung changes following thoracic irradiation. Acheva et al. present new data regarding the mechanism of RT-induced skin side effects focusing on the role of NFκB and Cox-2 in the generation of pro-inflammatory signals. Morini et al. investigate the impact of ionizing radiation on the permeability of the intestinal barrier in the context of colorectal cancer and show that several of the involved mechanisms are immune- and inflammation-related. Lumniczky et al. review radiation-induced immune and inflammatory reactions in the brain, highlighting potential mechanisms how these interactions can lead to long-lasting functional alterations and the development of cognitive impairment. Amelioration of chronic inflammation, induction of acute damage (e.g., tumor cell necrosis/danger), and counter-balancing the tumor- and radiation-derived immune suppression can in sum result not only in specific and long-lasting antitumor immunity but also in less frequent or less severe side effects and lead to an increased resistance toward radiation. This is highlighted in the paper by Singh et al. who show that the radioprotective effect of two well-characterized antioxidant compounds (podophyllin and rutin) is mainly immune-mediated.

Therefore, one of the beneficial effects of RT is, in some instance, to shift the equilibrium toward immune activation. For this, additional immunotherapy is mostly needed.

As discussed in their review by Wu et al., in addition to the direct effects of radiation on the different immune cell subsets, a key event in the (re)-activation of tumor-associated immune cells is the type of tumor cell death induced by radiation, as apoptosis, necrosis, autophagic cell death, and mitotic catastrophe differ in their ability to reverse immunosuppression and elicit these tumor-specific immune responses. As the release of metabolites such as inosine by dying or dead tumor cells can on the opposite stimulate the outgrowth of rare spared tumor cells (Chen et al.), the net outcome of radiation-therapy will depend on the competition between immunogenic and pro-tumorigenic events. Vaupel and Multhoff in particularly focus in their commentary on the role of adenosine as a consequence of hypoxia as metabolic immune checkpoint in the tumor microenvironment. Furthermore, the outcome of radiation exposure depends not only on the type of tumor and its microenvironment but also on the dose and quality of radiation and the irradiation scheme used. Even if more studies are required, especially on the effects of protons and carbon ions exposure (summarized by Ebner et al.), it is clear that these parameters can modulate the different aspects of the intercellular communication in the irradiated tumor microenvironment (reviewed by Diegeler and Hellweg). In addition to their direct effects on mature lymphocytes and T lymphocyte response to irradiation-induced bystander signals, ionizing radiation also affects T lymphocyte development. This aspect of the interactions between radiation and the immune system is addressed by Calvo-Asensio et al. in a research article where they analyze the response of thymic epithelial cells (TECs) to radiation exposure *in vitro* and *ex vivo*. TEC represent less than 1% of the cells found in the thymus, but these highly specialized cells are essential for the generation of mature, functional T lymphocytes. Although they are quite radio-resistant, the expression of many genes essential for proper T lymphocyte development is de-regulated after exposure. These effects probably contribute to the profound T cell deficiency observed in patient exposed to radiation in the frame of the conditioning regime before bone-marrow transplantation.

Last but not least, a special focus is placed on the investigation of low-dose radiation-induced immune mechanisms and inflammatory reactions. Szatmári et al. demonstrate in an *in vivo* experimental setup that extracellular vesicles are responsible for mediating certain radiation-induced bystander effects in the bone marrow. Erbeldinger et al. present a new method by which the effect of ionizing radiation on endothelial cells can be investigated *in vitro* where hemodynamical parameters are much closer to physiological conditions than using conventional cell cultures. In this system, they show that both low energy and heavily charged particles induce altered adhesion of peripheral blood lymphocytes and activation of the NFκB pathway. With the perspective of space travel and a Mars mission in a reasonably near future, the impact of cosmic radiation becomes a particularly important health problem, and the potential effects of charged heavy particles on the immune system and their long-lasting health consequences will need to be addressed. Since heavy particles are increasingly used in therapeutic radiation as well, their interaction with the immune system, in the view of a potential combination with immunotherapy should be carefully studied. These issues are dealt with in detail in the mini-review by Fernandez-Gonzalo et al.

Thus, the editors of the Research Topic hope that this collection of articles is able to give a good overview of the complex and often contradictory nature of the interactions between ionizing radiation and the immune system. Our intention was also to draw the attention of the non-radiobiological scientific community on these complex interactions and to highlight the fact that ionizing radiation is by far more than purely an immune suppressing agent. The increasing penetrance of low-dose ionizing radiation both through medical diagnostic or environmental sources or during cosmic travel in the population is becoming a major epidemiological concern world-wide and the mechanisms how low-dose radiation act and the potential long-term health consequences need to be thoroughly investigated.

## Author Contributions

All authors contributed in the editing of the Radiation and the Immune System Research Topic.

## Conflict of Interest Statement

The authors declare that the research was conducted in the absence of any commercial or financial relationships that could be construed as a potential conflict of interest.
